# *In vitro* reconstruction of human junctional and sulcular epithelium

**DOI:** 10.1111/jop.12005

**Published:** 2012-09-05

**Authors:** G Dabija-Wolter, V Bakken, M R Cimpan, A C Johannessen, D E Costea

**Affiliations:** 1 The Gade Institute, Faculty of Medicine and Dentistry, University of BergenBergen, Norway; 2 Biomaterials, Department of Clinical Dentistry, Faculty of Medicine and Dentistry, University of BergenBergen, Norway

**Keywords:** cytokeratins, differentiation, oral epithelia, organotypic, periodontal tissue

## Abstract

**BACKGROUND:**

The aim of this study was to develop and characterize standardized *in vitro* three-dimensional organotypic models of human junctional epithelium (JE) and sulcular epithelium (SE).

**METHODS:**

Organotypic models were constructed by growing human normal gingival keratinocytes on top of collagen matrices populated with gingival fibroblasts (GF) or periodontal ligament fibroblasts (PLF). Tissues obtained were harvested at different time points and assessed for epithelial morphology, proliferation (Ki67), expression of JE-specific markers (ODAM and FDC-SP), cytokeratins (CK), transglutaminase, filaggrin, and basement membrane proteins (collagen IV and laminin1).

**RESULTS:**

The epithelial component in 3- and 5-day organotypics showed limited differentiation and expressed Ki-67, ODAM, FDC-SP, CK 8, 13, 16, 19, and transglutaminase in a similar fashion to control JE samples. PLF supported better than GF expression of CK19 and suprabasal proliferation, although statistically significant only at day 5. Basement membrane proteins started to be deposited only from day 5. The rate of proliferating cells as well as the percentage of CK19-expressing cells decreased significantly in 7- and 9-day cultures. Day 7 organotypics presented higher number of epithelial cell layers, proliferating cells in suprabasal layers, and CK expression pattern similar to SE.

**CONCLUSION:**

Both time in culture and fibroblast type had impact on epithelial phenotype. Five-day cultures with PLF are suggested as JE models, 7-day cultures with PLF or GF as SE models, while 9-day cultures with GF as gingival epithelium (GE) models. Such standard, reproducible models represent useful tools to study periodontal bacteria–host interactions *in vitro*.

## Introduction

Anatomically, gingival epithelium consists of oral gingival epithelium (GE), sulcular epithelium (SE), and junctional epithelium (JE) 1. The JE mediates the attachment of gingiva to the teeth through hemidesmosomes and the internal basal lamina with a unique basement membrane (BM) composition, while being attached to the connective tissue by the external basal lamina, containing many of the components of gingival basement membrane, including collagen IV 2. JE is a multilayered epithelium with permeable structure, high turnover (the highest among oral tissues), and limited differentiation, lacking keratinization. The SE makes the transition between JE and GE and lines the gingival sulcus. The distinctive structure and function of these epithelia are also reflected by the presence of specific proteins and differentiation markers. The odontogenic ameloblast-associated (ODAM) protein 3 and follicular dendritic cell-secreted (FDC-SP) protein 4 have been recently identified as having a specific expression to JE. The expression of keratinocyte differentiation markers such as cytokeratins (CK), filaggrin, and transglutaminase 5–7 makes also a clear delimitation between these epithelia. CK are major structural proteins expressed by epithelial cells according with the stage of differentiation and the origin of the epithelium 8–10. Transglutaminase (TG) is an enzyme that plays an important role in the late differentiation of the mature keratinocyte 11, being expressed in oral tissues in spinous layers both in orthokeratinized (such as the hard palate) and non-keratinized (buccal mucosa) epithelia 12. Filaggrin is also a late marker of keratinization, present *in vivo* in orthokeratinized and parakeratinized areas of human oral epithelium 13,14.

The native GE was reported to express the cytokeratins 5 and 14 and occasionally CK19 in the basal layers. In the suprabasal layers, the native GE expresses CK 1/10 (characteristic for keratinized squamous stratified epithelia), CK 6/16 (markers of hyperproliferation), as well as transglutaminase and filaggrin 7,13. JE has a unique phenotype, expressing cytokeratins specific for simple epithelia, such as CK 7, 8, 18, and 19 and for basal layers (CK 5 and 14), but also for non-keratinizing stratified epithelia, CK 13 and 16 5,15–17. While expression of CK 8/18 in JE may vary in frequency and intensity 5,7, the presence of CK 19 is regarded as a consistent marker for JE 16,17. The SE is a non-keratinizing stratified squamous tissue lacking stratum granulosum, found in GE 18. SE displays higher number of cell layers and better differentiated than JE, but expresses CK 19, the marker of JE, to a lesser degree. The proliferation rate in SE is higher than in GE 19.

The SE and JE are continuously challenged by bacterial biofilm from subgingival plaque; thus, they represent crucial sites with respect to initiation and development of periodontal diseases. Bacteria can invade through epithelial layers, elaborate bacterial byproducts and induce a local immune response, which can lead to breakdown of periodontal tissues. Hence, *in vitro* studies investigating host–bacteria interactions mimicking periodontal conditions have more relevance to be performed by using culture models resembling JE or SE 20. *In vivo*, the phenotype of the epithelium and implicitly the CK expression are influenced by the interactions with the sub-epithelial mesenchyme and eventual pathological changes (such as inflammation, dysplasia or malignant transformation of epithelia) 21–23. Similarly, when oral tissues are reconstructed *in vitro*, as multilayered three-dimensional (3D) organotypic (OT) culture models, the CK expression of the epithelial component is strongly influenced by the type of underlying matrix and the origin of fibroblasts contained in it 8,24,25.

The aim of this study was thus to develop and characterize standardized models of junctional and sulcular human epithelium that can be used to study periodontal bacteria–host tissue interactions. For this purpose, the morphology, cell proliferation, and CK profile of OT models reconstructed with primary human gingival keratinocytes on top of fibroblast-populated collagen biomatrices were assessed at different stages of growth. The impact of two different types of oral fibroblasts (periodontal vs. gingival) on epithelial differentiation and phenotype was evaluated. We have investigated which combination of type of fibroblasts and growth stage may result in a model that resembles best the junctional or sulcular epithelium. Such model may represent a functional tool for studying periodontal bacteria–host tissue interactions *in vitro*.

## Materials and methods

### Materials

Dulbecco's modified Eagle's medium (DMEM) was acquired from Sigma (St Louis, MO, USA). Serum-free keratinocyte medium (KSFM), human recombinant epidermal growth factor (EGF), bovine pituitary extract (BPE), fetal bovine serum (FBS), l-glutamine, penicillin, streptomycin, and amphotericin B were acquired from GibcoBRL (Grand Island, NY, USA). Cell culture flasks and plates were from Nunc (Napervile, IL, USA), and center-well organ culture dishes were from BD Biosciences (San Jose, CA, USA). The Envision+ system used in immunohistochemistry was from DAKO (Glostrup, Denmark). Antibodies used in this study are presented in Table 1.

**Table 1:** Immunohistochemistry: information on secondary antibodies used

Antibody	Type	Clone/number	Titration	Source
FDC-SP	Rabbit polyclonal	Anti-C4 or f7 HPA014326	1:80	Sigma, St Louis, MO, USA
ODAM	Rabbit polyclonal	HPA036543	1:10	Sigma, St Louis, MO, USA
CK 8	mouse monoclonal	Supernatant	1:5	CRUK
CK 10	IgG-1	–	1:50	DAKO A/S, Glostrup, Denmark
CK 13	IgG-1	KS-1A3	1:50	Novocastra
CK 16	Mouse monoclonal	Supernatant	1:5	CRUK
CK 19	Mouse monoclonal	Supernatant	1:5	CRUK
Ki-67	IgG-1	MIB-1	1:25	DAKO A/S, Glostrup, Denmark
Filaggrin	IgG-1	15CID	1:50	Monosan
TG	Rabbit polyclonal	pab0061	1:250	Covalab-UK
Collagen IV	IgG-1	CIV 22	1:50	DAKO A/S, Glostrup, Denmark
Laminin-1	IgG-2a	4C7	1:1200	DAKO A/S, Glostrup, Denmark

CRUK, Cancer Research United Kingdom (Abs were a kind gift from Prof. I. C. Mackenzie, Institute for Cell and Molecular Science, London, UK).

### Cell isolation and culture

Clinically healthy adult volunteers (*n* = 12) undergoing surgical removal of wisdom teeth were informed about the purpose of study and gave their written consent regarding sample collection. The study was approved by the Regional Committee for Medical Ethics in Research. A total of 12 samples of gingival mucosa showing no sign of clinical inflammation at collection time and seven wisdom teeth were used to generate primary keratinocytes and fibroblasts. Samples of native normal tissues of JE (*n* = 6), SE (*n* = 6), and GE (*n* = 7) served as controls for *in vivo* expression patterns of the investigated proteins. The normal/native tissues and specimens were previously collected with ethical clearance by one of the co-authors for characterization of periodontal tissue. Normal human gingival mucosa, skin, breast, tonsil, and prostate specimens served as positive controls for protein detection. Gingival epithelial cells (GECs) were isolated as previously described through a combination of enzymatic digestion and mechanical separation of cells and cultured in serum-free media (KSFM) supplemented with 1 ng/ml EGF, 25 μg/ml BPE, 20 μg/ml l-glutamine, 100 U/ml penicillin, 100 μg/ml streptomycin, and 0.25 μg/ml amphotericin B 26. We have previously published the protocol for the isolation and characterization of gingival fibroblasts (GF) and periodontal ligament fibroblasts (PLF) based on alkaline phosphatase (ALP) expression as marker of differentiation 27. Briefly, fibroblast cells were cultured in DMEM supplemented with 10% FBS, 20 μg/ml l-glutamine, 100 U/ml penicillin, 100 μg/ml streptomycin, and 0.25 μg/ml amphotericin. The cells used were GECs in their 1st to 3rd passage and GFs or PLFs in their 2nd to 5th passage. All cultures including the organotypic models were kept in a humidified atmosphere at 37°C and supplemented with 5% CO_2_.

### Construction and harvesting of OT tissue models

In our laboratory, the construction of an OT model of oral mucosa that reaches its maturity at day 9 of development after adding the keratinocytes on top of fibroblast-populated biomatrices has been well established and previously described 28. The multilayered epithelium was elaborated using GECs grown on top of collagen matrices populated with oral fibroblasts. In this study, 250 000 fibroblast cells, either GF or PLF (in some cases matched from the same donor), were used for each collagen matrix. A number of 500 000 GECs in 1-ml KSFM were seeded on top of each collagen matrix the following day, considered day 1. The further handling of OTs was carried out as previously described 28, and the tissues being placed on grids and lifted on the air-liquid interface. The harvesting of the OTs was performed at different time intervals of their development: at day 3, 5, 7, and 9. The OT tissue samples further investigated here were formalin-fixed and paraffin-embedded. Six different experiments with GF/ PLF were carried out.

### Tissue preparation, staining, and analysis

Sections of 5 μm thickness from formalin-fixed OT tissues were cut and stained with hematoxylin–eosin (HE) or used for immunohistochemistry (IHC). IHC staining was carried out as routinely in our laboratory and previously described. Information on the primary antibodies used in the study is detailed in Table 1. All primary antibodies were applied for 60 min. TE buffer (10 mM Tris, 0.1 mM EDTA, pH 9) was used for all markers except for TG (where citrate buffer pH 6 was used) and 60-min incubation. As positive controls were used tissue samples of breast epithelium and prostate for markers CK 8 and 19; gingival mucosa, skin, and tonsil tissue for ODAM, FDC-SP, collagen IV and laminin-1; buccal mucosa for CK 13; and palate epithelium for CK 10, CK 16, transglutaminase and filaggrin. Specimens incubated with antibody diluent only (DAKO) or isotype-matched antibody (DAKO) instead of primary antibody were used as a negative control.

The HE stained tissues were evaluated under a light microscope (Leica DMLM, GmbH, Munster, Germany). Epithelial cell layers were counted and evaluated morphologically for epithelial growth, degree of keratinization, and differentiation pattern and compared with control tissues. Exfoliated cells were not included in the counting of cell layers, and the tissue ends (margins) were also excluded 29,30. Each tissue was examined with a 40× objective, and images of three to six fields separated by 334 μm distance were taken with a camera connected to a computer. By use of Olympus Soft-DP 5.0 software package (Soft Imaging System GmbH), histomorphometric and immunohistochemical analyses were performed on each image, which had a horizontal width of 334 μm at this magnification.

For quantification, the Ki-67- and CK19-expressing cells and also the negative cells were counted manually at 400-fold magnification. The Ki-67 immunopositive cells were counted in all layers in sections for OTs harvested at day 3, and starting from day 5 up, they were counted separately in basal/parabasal and suprabasal regions of the epithelial component. The delimitation of layers in basal and suprabasal regions was made according to morphology of the cells in the respective layers (higher ratio nucleus/cytoplasm, vertical or angular orientation of cell axis on the basal membrane). Cell proliferation was calculated as percentage of Ki-67-positive cells from all cells of the basal layers and/or the total number of Ki-67 expressing nuclei per 334 μm length of the epithelial–connective tissue equivalent interface. The number of proliferating nuclei in suprabasal layers was also registered. The CK 19-expressing cells were calculated as the percentage of positive cells within all cell layers from day 3 to day 9.

The *in vitro* constructed models were compared at different development stages or related to the type of fibroblasts used in the matrix and also with the control tissues.

### Statistical analysis and data presentation

Each individual experiment was run in duplicate or triplicate. The mean of CK 19 or Ki-67-expressing cells was calculated per experiment, and paired *t-test* was performed to compare the GF vs. PLF tissues within the same experiment at each stage of differentiation. Data were analyzed for other parameters using analysis of variance (ANOVA) with Bonferroni multiple comparisons using SPSS version 13 statistical software package (SPSS Inc, Chicago, IL, USA). *P*-values <0.05 were considered significant.

## Results

### Histomorphometric evaluation of the epithelial compartment in reconstructed tissues

**Figure 1:**
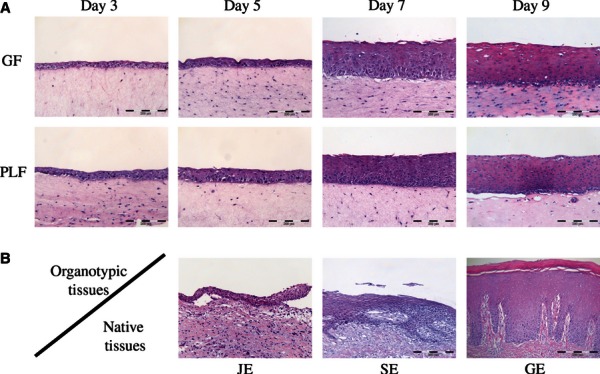
Hematoxilin–eosin stained tissues. (A) Organotypic models constructed with gingival epithelial cells grown on top of collagen matrices populated with GF (gingival fibroblasts) or PLF (periodontal ligament fibroblasts) harvested at different stages of growth. (B) Native tissues used as controls. Scale bar: 200 μm.

At day 3 of development, the epithelium was comprised of 1–4 cell layers (37.73 ± 10.87 μm thickness, mean, and standard deviation) without any sign of differentiation in all OTs. At day 5, the epithelial component displayed 3–5 cell layers (49.79 ± 11.50 μm) and very little differentiation, as only one superficial cell layer had a more flatten morphology and could be considered as suprabasal. For both day 3 and day 5 OTs, there was no noticeable difference between OTs constructed using GF or PLF, as assessed by histological analysis of HE stained tissues (Fig. 1).

At day 7, the OT models had an epithelial component of 9–13 layers (130.93 ± 29.28 μm), increasing to 11–16 layers at day 9 (190.83 ± 16.78 μm). In all 7- and 9-day OT models, the differentiation was visible and the presence of the spinous layers could be noticed. No significant difference in the thickness could be observed between OTs constructed with GF or PLF at days 7 and 9 either. Variations from batch to batch could be observed regarding size of the cells, total number of layers or degree of differentiation. However, some of the tissues constructed with GF showed a well-differentiated superficial cell layer compared with those constructed with PLF at day 9, but such differences were not found consistently in all batches.

### Distribution and quantification of proliferating cells (Ki-67)

**Figure 2:**
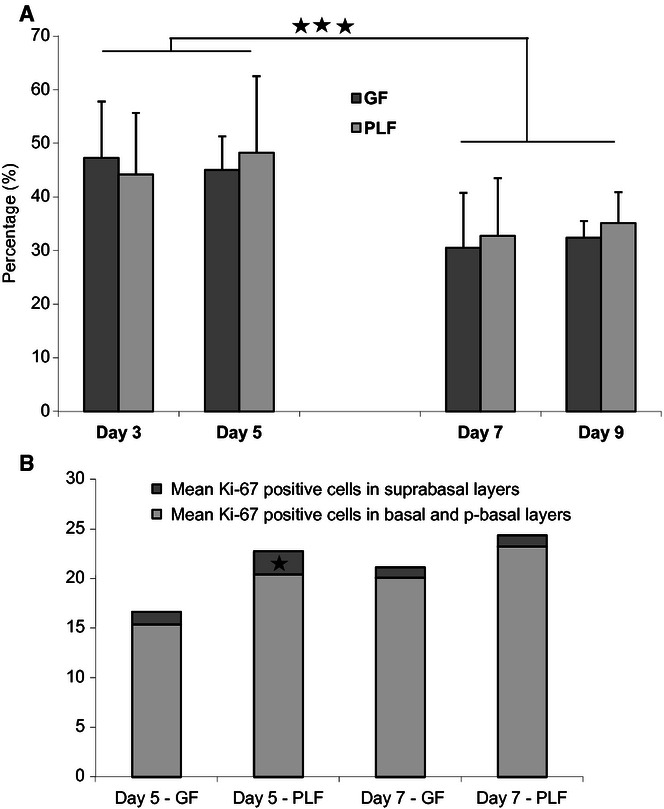
Quantification and distribution of proliferating cells (Ki-67 positive) in organotypic models. (A) Percentage of Ki-67-positive cells in basal and parabasal layers; (B) Mean (*n* = 4 independent experiments) of Ki-67-positive cells per 334 μm length in basal (light gray bars) and suprabasal (dark gray bars) layers. Statistical significance: ***P < 0.001; *P < 0.05. GF, gingival fibroblasts; PLF, periodontal ligament fibroblasts.

*In vivo*, the cell proliferation rate is an important feature that differentiates JE, having the highest cell turnover among all types of periodontal and oral tissues. Therefore, we have quantified the Ki-67-expressing cells in the reconstructed OT models for the purpose of comparison. The distribution and number of Ki-67-positive cells varied in culture with the time allowed for development and differentiation. Day 3 and day 5 OT cultures showed proliferating cells distributed in all cell layers, similarly with the situation encountered in JE *in vivo*. In these OTs, we found significantly higher proliferation rate (*P* < 0.001), in cultures both with GF and with PLF, compared with those harvested at later time points (day 7 and 9) (Fig. 2A). The type of fibroblasts was not found to influence significantly (*P* = 0.38) the proliferation rate within the basal and parabasal layers of organotypic models. Interestingly, significant more Ki-67-positive cells were detected at day 5 in suprabasal layers (*P* = 0.018, paired *t*-test) in tissues with PLF (2.34 ± 0.39, mean and standard error) than in those with GF (1.28 ± 0.43). At day 7, fewer Ki-67-expressing nuclei were counted in suprabasal layers, (1.03 ± 0.51 in GF OTs and 1.13 ± 0.59 in those with PLF, respectively; Fig. 2B). At day 9, no Ki-67-expressing cells were present in suprabasal layers.

### Expression of specific markers of JE

**Figure 3:**
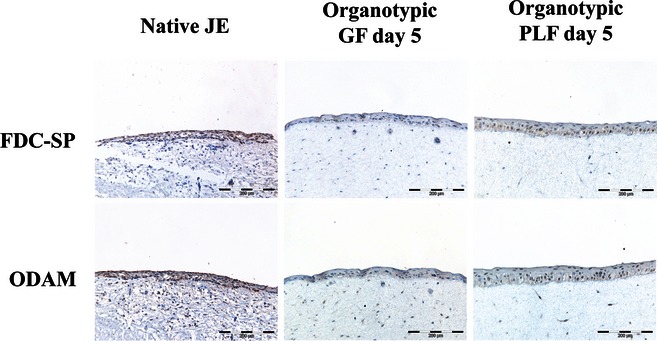
Specific markers of JE: ODAM and FDC-SP in OT models of day 5 constructed with GF and PLF and in native JE. Scale bar: 200 μm.

The JE-specific markers ODAM and FDC-SP were detected in all epithelial layers of day 3 and day 5 cultures. Both GF and PLF supported the expression of these two markers in the suprajacent epithelium, but PLF stronger than GF (Fig. 3). The expression of ODAM was maintained at day 7 and 9 in basal cell layer only, while FDC-SP was not detected in OT models of day 7 and 9. ODAM was expressed in all layers of native JE and SE, but in SE the staining was stronger toward the basal and superficial layers. In native GE, expression of ODAM was maintained in the basal layer with only few scattered positive cells in the superficial layers. In the native JE, 50–75% of cells of all layers stained positive for FDC-SP. The staining faded toward SE and GE. Nevertheless, in 50% of the cases examined, the expression of FDC-SP was maintained in the spinous layer of GE, although much less intense than in JE.

### Expression pattern of differentiation markers in reconstructed and control tissues

A summary of expression of the differentiation markers, both in reconstructed tissues and in controls, is presented in Table 2. The CK 13, 16, and 19 were consistently found expressed in the epithelium of all OT cultures, constructed either with GF or with PLF. In OTs, the marker for stratified squamous non-keratinized epithelia CK 13 was present throughout all epithelial layers at day 3 but more variable at day 5, becoming stronger in upper layers. At days 7 and 9, the expression of CK 13 became polarized, as all cells of suprabasal layers stained strongly for this marker, while cells in basal layers were positive in a variable proportion from a few scattered cells to about 50% positive. Slight variations were observed from batch to batch or even within the same tissue model. The CK 13 staining in JE control tissue was positive in all layers, in a proportion slightly varying between samples, from 85% to 100%. The SE control tissues expressed CK 13 only in suprabasal layers, and the staining was gradually lost toward GE, where this marker was nearly absent.

**Table 2:** Results of immunostaining for a panel of different markers in control tissues and organotypic models. The presence (+) or absence (−) of immunopositive cells or their presence in only some of the samples (+/−) is marked in the table without indicating the intensity of staining or any quantification

	In organotypics^a^					Control SE		
				
Marker	d3	d5	d7	d9	Control JE	AM	GM	Control GE
FDC-SP	+	+	−	−	+	+/−	+/−	+/−
ODAM	+	+	+/−	+/−	+	+	+	+/−
CK 8	+	−	−	−	−	−	−	−
CK 10	−	−	+/−	+	−	−	+	+
CK 13	+	+	+	+	+	+	−	−
CK 16	+	+	+	+	+/−	+/−	−	+
CK 19	+	+	+	+	+	+	+	+
Filaggrin	−	−	−	−	−	−	−	+
TG	+	+	+	+	+	+	+	+
Collagen IV	−	+/−	+	+	+	+	+	+
Laminin-1	−	+/−	+	+	+	+	+	+

a The description of the immunostaining refers to both GF and PLF – constructed organotypics if not otherwise mentioned. AM, apical margin, GM, gingival margin.

At day 3, the OTs expressed CK 16 in the cells of the upper layers and sparsely in the basal cell layer, where only a few positive cells were found, matching with the expression of this marker determined in control tissues. In JE, CK 16 was found positive only in half of the samples where was expressed by approximately 30% of cells. OT tissues constructed with both GF with PLF from day 5 to day 9 stained positive for CK 16 only in the suprabasal layers. SE control tissues displayed CK 16 immunopositive cells in the spinous layer and sparsely in basal cells and the staining faded or disappeared toward JE. In GE control tissues, we have found CK 16-positive cells in parabasal and suprabasal cells, much stronger stained in the spinous layer.

**Figure 4:**
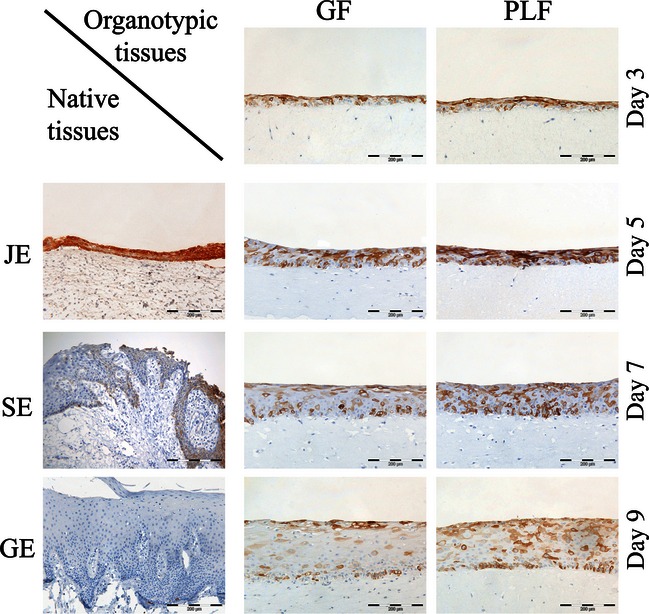
CK 19 staining in organotypics and in control tissues. Scale bar: 200 μm.

CK 19 was expressed in all OT reconstructed tissues, in all cell layers, stronger at 3 and 5 days of culture and slightly fading at days 7 and 9, especially in OTs constructed with GF (Fig. 4). The percentage of CK 19-positive cells decreased gradually with the time allowed for maturation of the tissue cultures. In GF tissues, from 70.3 ± 2.83% (mean and standard error) CK19-positive cells determined at day 3 and 67.91 **±** 2.03% at day 5, the percentage decreased further to 53.17 **±** 2.65% at day 7 and 31.24 **±** 4.97% at day 9. The length of time the *in vitro* reconstructed tissues were allowed to develop in culture before harvesting and analyzing was found statistically significant by ANOVA test for repeated measurements (*P* = 0.003) as having the main effect for expression of this marker in OT culture. The OTs constructed with PLF expressed CK 19 in a higher percentage than those with GF, as 76.95 ± 0.87% of the cells at day 3 stained positively and 72.81 ± 2.72% at day 5, respectively. The proportion of CK 19-expressing cells decreased further, reaching 56.29 ± 2.04% at day 7 and 35.45 ± 5.44% at day 9 in PLF organotypics. Although the expression of CK 19 was maintained higher in PLF than in GF organotypics throughout all development stages, when these groups were compared by use of paired *t*-test, statistical significance was found only at day 5 (*P* = 0.047). *In vivo*, CK 19 was expressed intensely in all samples in all layers of JE, while in control SE the expression changed from intensely positive cells toward apical margin, until almost disappearing toward the coronal region. In GE, there were very few CK 19-positive cells, scattered in the basal layers in rete ridges (Fig. 4).

The marker for simple epithelia CK 8 was weakly positive in few of our OT cultures constructed with both GF and PLF, only at day 3 and not in cultures at day 5 or older. The control tissues did not stain positive for CK 8 either, but few single cells in the basal layers of rete ridges in GE were observed, as for CK 19. The positive control (prostate tissue) showed intense staining for CK 8.

In OT cultures grown to day 3 and 5, the marker for stratified keratinized epithelia CK 10 was absent. In samples harvested at day 7, CK10-positive cells were found sparsely in spinous layers, in approximately 20% of the samples, in OT tissues constructed with both GF and PLF. The expression of CK 10 OTs was much stronger at day 9, in spinous and superficial layers, in cultures with both types of fibroblasts. This pattern was observed also in control tissues: absence of CK 10 in JE, very rare positive cells in SE (more toward gingival margin), and strong expression in spinous and superficial layers of GE.

Transglutaminase stabilizes the epithelium by cross-linking constituent proteins; thus, it has been used as a marker for epidermal cell maturation. It was expressed in all layers of day 3 cultures, constructed with either GF or PLF, while in days 5, 7, and 9 it was present mainly in the upper layers and seemed stronger in OT with GF. In control JE, transglutaminase was found positive in the upper part of the tissue. In SE, the staining faded toward apical margin, but it was stronger in spinous layers toward gingival margin. Transglutaminase was expressed in spinous layers of GE control tissues.

The other marker of late keratinisation, filaggrin, was not found positive in any of OT cultures. This marker lacked as well from JE and SE control samples, but was found positive in upper layers of GE control tissues.

**Figure 5:**
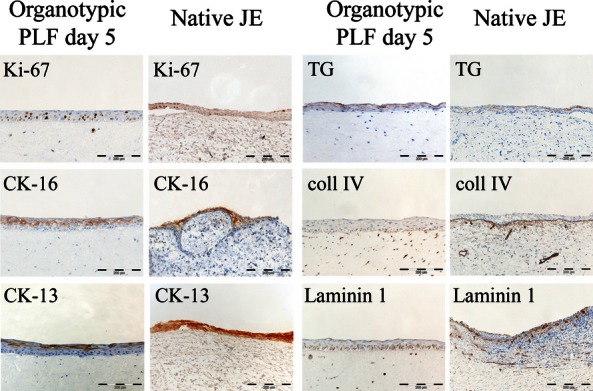
Immunohistochemistry showing similarities in protein expression found between a representative organotypic culture, PLF – day 5, and control JE. Scale bar: 200 μm.

Collagen IV, characteristic for basal membranes and external basal lamina in JE, was not detected in OTs of day 3. At day 5, collagen IV was found sparsely deposited in OTs constructed with both GF and PLF (Fig. 5). The deposition of collagen IV was still weak and irregular at day 7, but was observed as a continuous line in OTs grown till day 9. Laminin-1, another protein characteristic for basal membranes, was expressed by the epithelial cells nearby epithelium-collagen matrix interface in all OTs, but its deposition extracellularly was not detected till day 5, when it was sparce and discontinuous in OTs with both GF and PLF (Fig. 5). The deposition of laminin-1 became more obvious at day 7 and further more developed at day 9, when it was visible as a continuous line in OTs constructed with both type of fibroblasts.

Concluding, based on the analyses of tissue morphology and differentiation, correlated with the expression pattern of different markers, and the decline of proliferating cells in the basal layers, the OT cultures could be grouped in ‘early’ (day 3 and 5) and ‘late’ (day 7 and 9) culture models. The ‘early’ OT cultures had low number of epithelial layers, none, or very little differentiation (one suprabasal layer at day 5) and high number of proliferating cells distributed in all cell layers. The specific markers (FDC-SP and ODAM), the CKs (CK 19, 13, 16, and 8), and transglutaminase normally expressed by native JE 5,7,15,17 were also determined in our ‘early’ OT models (especially in those constructed with PLF) as having a distribution pattern similar with the one encountered *in vivo*. The markers CK 10 and filaggrin normally lacking from JE 7,13,16,24 were also lacking from day 3 and 5 OT models. Deposition of basement membrane proteins was detected only from day 5 OTs.

## Discussion

In the present study that aimed to develop and characterize *in vitro* models for periodontal tissue, we found that depending on the time allowed for the model to mature, we can develop models that resemble junctional, sulcular, or gingival epithelium by using gingival keratinocytes and periodontal and gingival fibroblasts.

Based on these findings, we suggest that the OT tissues grown for 5 days could serve as *in vitro* JE models. Particularly, the OTs grown with PLF showed a better preservation of the JE phenotype, reflected by a stronger expression of specific markers FDC-SP and ODAM, and higher percentage of CK 19-positive cells and proliferating cells in the suprabasal cell layers (Fig. 4). Although these models lack the internal basal lamina, they show some deposition of components of the external basal lamina (collagen IV and laminin 1). We propose that day 7 OT cultures could be taken in consideration as a model for SE, because of a higher number of cell layers, limited differentiation, presence of proliferating cells in suprabasal layers, and the expression pattern of different CKs and basement membrane proteins similar to the SE. The OT model containing GF matured to day 9 may be used as culture model for GE, as we have already indicated in a previous study 31.

Both the time in culture and type of fibroblasts influenced the tissue morphology, proliferation, and differentiation, but only time in culture had a significant impact on expression of CK 19 in our OT models. Our results are in agreement with those of Locke et al. 24 who reported that the type of fibroblasts used in the connective tissue equivalent influenced the CK expression in the epithelial component, but did not induce a complete change of the phenotype. However, they reported a markedly less growth of the epithelial component and weaker expression of CK 10 in the OT cultures engineered with PLF, compared with those with GF, having more cell layers and stronger CK 10 expression. In our work, we have observed higher differentiation in mature (day 9) OTs constructed with GF than in those with PLF; but the variations in epithelial thickness were not found significant at any time point (data not shown). These differences might be explained by different culture conditions, *for example,* time allowed for maturation, different culture medium, and different number of fibroblasts used in these two studies. While the study of Locke et al. investigates the tissues at one time point only, we have looked at different steps of development. In addition, we present here a feasible model of JE that can be obtained from GECs, which are easier to obtain and grow in culture in comparison with JE cells, as suggested by Locke et al. The novelty of our study is that by using the same cell type, namely GECs, one can build models of JE, SE, and GE by modulating the time in culture instead of using cells with originally different phenotype. Thus, the use of GECs instead of JE cells might present significant logistic advantages for constructing models for use as biological tools in experimental setting.

We have shown here that CK 19 was positive in all OT tissues up till day 9, even if these were constructed with GF. This differentiation marker is distinctive for JE but is sparsely expressed also in GE, and it appears to be increased in periodontitis 21,23,32,33. It has been shown that when cultured *in vitro*, cells isolated from GE were also found to express CK19, similarly with those isolated from JE 34. Gain of CK 19 in the gingival epithelial cells grown in culture both in colonies (monolayers) and in multilayered OT models was reported also by other studies 24,35. These studies did also point out to a slight change in the phenotype of gingival epithelial cells cultured *in vitro*, expressing markers of differentiation such as CK 1, 10, and 13 less intensely than *in vivo* and at the same time ‘gain’ or increase in intensity of the expression of cytokeratins characteristic for simple epithelia, CK 8/18 and 19, and for high proliferating tissues (CK 6/16). We also report here a stronger staining for CK 16 and the presence of CK 8 only in day 3 reconstructed tissues, compared with *in vivo* samples, where these markers were found scarce or absent. In conclusion, we agree with the above-mentioned studies that expression of differentiation markers in reconstructed culture models is close to the *in vivo* pattern, but not identical.

However, in the present study, we show a steady decline in time in expression of ODAM, FDC-SP and in the number of CK19-positive cells in OT cultures and determined that time in culture had a significant impact on expression of these markers. This finding indicates once more that 3D organotypic models are more appropriate experimental models and closer to the *in vivo* situation than 2D culture models. The development of such serial models is justified by the continuous and increasing interest of studying interactions between oral bacteria, including putative periodontal pathogens, and human oral tissues 31,36–40.

In our study, a higher expression of ODAM, FDC-SP, and CK 19 in the epithelial compartment was at all time points better supported by PLF than GF, although the type of the fibroblasts used in the connective tissue equivalent became statistically significant for expression of CK19 just at one time point, at day 5.

Of note is that at the same development point (day 5), the presence of PLF in the matrix influenced significantly the distribution pattern of the proliferation cells in the epithelium, PLF supporting suprabasal proliferation.

In conclusion, this study identifies certain culture conditions (time points and type of fibroblasts) that can be reproducibly used to develop standardized (serum free) models of human junctional or sulcular epithelium. Based on the characterization of the tissues we made, we suggest the use of the model with PLF at day 5 as a JE model for bacterial–host cell interactions. As for SE model, we consider appropriate the OT cultures constructed with either PLF or GF grown to day 7; and those with GF at/after day 9 as suitable for a gingival model. Such models may constitute a very useful working tool in investigating the mechanisms involved in periodontium breakdown in periodontal disease, especially in host tissue – bacterial interaction studies.
